# Supplementary material for: Modulatory effects of noradrenergic and serotonergic signaling pathway on neurovascular coupling
Source: Commun Biol. 2024 Mar 8;7:287. doi: 10.1038/s42003-024-05996-y (PMC10923894; doi:10.1038/s42003-024-05996-y)
Supplement: Supplementary file 2 — Reporting Summary [file 42003_2024_5996_MOESM2_ESM.pdf]

nature portfolio

Corresponding author(s): Cam Ha T. Tran

Last updated by author(s): 2024/02/09

Reporting Summary

Nature Portfolio wishes to improve the reproducibility of the work that we publish. This form provides structure for consistency and transparency in reporting. For further information on Nature Portfolio policies, see our Editorial Policies and the Editorial Policy Checklist.

Statistics

For all statistical analyses, confirm that the following items are present in the figure legend, table legend, main text, or Methods section.

n/a	Confirmed	
☐	☒	The exact sample size (*n*) for each experimental group/condition, given as a discrete number and unit of measurement
☐	☒	A statement on whether measurements were taken from distinct samples or whether the same sample was measured repeatedly
☐	☒	The statistical test(s) used AND whether they are one- or two-sided<br>*Only common tests should be described solely by name; describe more complex techniques in the Methods section.*
☒	☐	A description of all covariates tested
☐	☒	A description of any assumptions or corrections, such as tests of normality and adjustment for multiple comparisons
☐	☒	A full description of the statistical parameters including central tendency (e.g. means) or other basic estimates (e.g. regression coefficient) AND variation (e.g. standard deviation) or associated estimates of uncertainty (e.g. confidence intervals)
☐	☒	For null hypothesis testing, the test statistic (e.g. *F*, *t*, *r*) with confidence intervals, effect sizes, degrees of freedom and *P* value noted<br>*Give P values as exact values whenever suitable.*
☒	☐	For Bayesian analysis, information on the choice of priors and Markov chain Monte Carlo settings
☒	☐	For hierarchical and complex designs, identification of the appropriate level for tests and full reporting of outcomes
☒	☐	Estimates of effect sizes (e.g. Cohen's *d*, Pearson's *r*), indicating how they were calculated

Our web collection on statistics for biologists contains articles on many of the points above.

Software and code

Policy information about availability of computer code

Data collection	Our data were collected using Thorlabs Bergamo II and ThorImage or Olympus FV1000 confocal microscope, and FV10-ASW
Data analysis	We used Fiji/ImageJ to process all our data and GraphPad Prism for all statistical analyses.

For manuscripts utilizing custom algorithms or software that are central to the research but not yet described in published literature, software must be made available to editors and reviewers. We strongly encourage code deposition in a community repository (e.g. GitHub). See the Nature Portfolio guidelines for submitting code & software for further information.

Data

Policy information about availability of data

All manuscripts must include a data availability statement. This statement should provide the following information, where applicable:

- Accession codes, unique identifiers, or web links for publicly available datasets
- A description of any restrictions on data availability
- For clinical datasets or third party data, please ensure that the statement adheres to our policy

All experimental data are provided in the manuscript. Source data are provided in DRYAD. https://doi.org/10.5061/dryad.hqbzkh1q7

Research involving human participants, their data, or biological material

Policy information about studies with human participants or human data. See also policy information about sex, gender (identity/presentation), and sexual orientation and race, ethnicity and racism.

Reporting on sex and gender	N/A
Reporting on race, ethnicity, or other socially relevant groupings	N/A
Population characteristics	N/A
Recruitment	N/A
Ethics oversight	N/A

Note that full information on the approval of the study protocol must also be provided in the manuscript.

Field-specific reporting

Please select the one below that is the best fit for your research. If you are not sure, read the appropriate sections before making your selection.

☒ Life sciences ☐ Behavioural & social sciences ☐ Ecological, evolutionary & environmental sciences

For a reference copy of the document with all sections, see nature.com/documents/nr-reporting-summary-flat.pdf

Life sciences study design

All studies must disclose on these points even when the disclosure is negative.

Sample size	Sample sizes were chosen to give statistically significant data according to the standard experiments in the field.
Data exclusions	Some data was excluded due to too much movement from the animals, which introduced motion artifact.
Replication	Experiments were replicated for at least 3 trials/vessel in all in vivo two-photon imaging experiments.
Randomization	Animals were randomly assigned to control or treatment groups. If it was possible, we performed both control and treatment on the same animal.
Blinding	For most of the awake in vivo two-photon imaging, we can't use blinding as both control and treatment were performed on the same animal. For the immunohistochemistry experiments, the experimenters did not know which animal was control and which animal was treated.

Reporting for specific materials, systems and methods

We require information from authors about some types of materials, experimental systems and methods used in many studies. Here, indicate whether each material, system or method listed is relevant to your study. If you are not sure if a list item applies to your research, read the appropriate section before selecting a response.

Materials & experimental systems

n/a	Involved in the study	
☐	☒	Antibodies
☒	☐	Eukaryotic cell lines
☒	☐	Palaeontology and archaeology
☐	☒	Animals and other organisms
☒	☐	Clinical data
☒	☐	Dual use research of concern
☒	☐	Plants

Methods

n/a	Involved in the study	
☒	☐	ChIP-seq
☒	☐	Flow cytometry
☒	☐	MRI-based neuroimaging

Antibodies

Antibodies used	rabbit anti-dopamine beta hydroxylase (Abcam ab 209487; goat anti-5-HT (Abcam ab66047)

Validation	ab 209487 has been referenced in more than 10 recently published articles.<br>ab66047 has been referenced in more than 20 articles published in the past 3 years.

Animals and other research organisms

Policy information about studies involving animals; ARRIVE guidelines recommended for reporting animal research, and Sex and Gender in Research

Laboratory animals	We used Aldh1l1Cre-ERT2xGCaMP6f mice.
Wild animals	N/A
Reporting on sex	These findings are from male mice.
Field-collected samples	N/A
Ethics oversight	All animal procedures were approved by the Animal Care and Use Committee of the University of Nevada, Reno.

Note that full information on the approval of the study protocol must also be provided in the manuscript.

Plants

Seed stocks	N/A
Novel plant genotypes	N/A
Authentication	N/A
